# Diagnostic and treatment guidelines for gastrointestinal and genitourinary endometriosis

**DOI:** 10.4274/jtgga.2017.0143

**Published:** 2017-12-15

**Authors:** Stacy Young, Megan Kennedy Burns, Lucia DiFrancesco, Azadeh Nezhat, Camran Nezhat

**Affiliations:** 1 Camran Nezhat Institute and Center for Special Minimally Invasive and Robotic Surgery, California, USA; 2 Stanford University Medical Center, California, USA; 3 University of California, San Francisco, School of Medicine, San Francisco, USA

**Keywords:** Laparoscopy, endometriosis, individualization, general surgery, gynecology

## Abstract

Endometriosis is commonly misdiagnosed, even among many experienced gynecologists. Gastrointestinal and genitourinary endometriosis is particularly difficult to diagnose, and is commonly mistaken for other pathologies, such as irritable bowel syndrome, interstitial cystitis, and even psychological disturbances. This leads to delays in diagnosis, mismanagement, and unnecessary testing. In this review, we will discuss the diagnosis and management of genitourinary and gastrointestinal endometriosis. Medical management may be tried first, but often fails in cases of urinary tract endometriosis. This is particularly important in cases of ureteral endometriosis because silent obstruction can lead to eventual kidney failure. Thus, we recommend complete surgical treatment in these cases. Bladder endometriosis may be managed more conservatively, and only if symptomatic, because these rarely lead to significant morbidity. In cases of bowel endometriosis, we recommend medical management first in all cases, and the least invasive surgical management only if medical treatment fails. This is due to the extensive nervous and vasculature supply to the lower rectum. Injury to these nerves and vessels can cause significant complications and postoperative morbidity.

## INTRODUCTION

Endometriosis affects up to 10% of all reproductive-age women, and affects approximately 35-50% of women with pelvic pain and infertility (1). It can manifest as deeply infiltrative endometriosis (DIE) or superficial lesions of the peritoneum and serosa. Endometriosis predominantly affects the pelvic reproductive organs, but can also be found in non-reproductive organs, known as extragenital endometriosis. The most common site of extragenital endometriosis is the gastrointestinal and urinary tract (2). Gastrointestinal endometriosis is found in 3.8-37% (3,4) of women with a known diagnosis of endometriosis, and urinary tract endometriosis is found in 1-6% of these women (2). The wide range reported in the literature is likely due in part to the difficulty in diagnosis of this enigmatic disease.

## PATHOGENESIS

There are numerous theories regarding the pathophysiology of endometriosis. One of the earliest and most instinctive theories proposed is that of retrograde menstruation, which describes the retrograde spillage of menstrual blood from the fallopian tubes during menstruation. Endometrial cells implant in the peritoneal cavity causing pain, inflammation, and fibrosis. Some observations supporting the retrograde menstruation theory are that;

1) Women with genital tract obstructions are more likely to have endometriosis (5),

2) The distal ureter is affected more often than the proximal ureter, possibly due to its closer proximity to the uterus and dependent location (6),

3) The left ureter is affected more commonly than the right (7,8), which may be due to obstruction of peritoneal flow by the sigmoid colon on the right (9,10),

4) Bladder endometriosis is found less commonly in women with a retroverted uteruses, potentially due to the larger space for endometrial cells to disseminate rather than implant on the bladder (11).

However, this theory is not all encompassing because 90% of women have retrograde menstruation, but only 10% of women develop endometriosis. Thus, further steps are necessary for endometrial cells to transform into endometrial implants (9,12). The second theory has an immunologic basis. It has been observed that there is a high incidence of endometriosis in women with autoimmune disorders, such as systemic lupus erythematosus, thyroid disease, 4 rheumatoid arthritis, Sjogren syndrome, asthma, and eczema (13). Dysregulation of the immune system may prevent normal clearance of ectopic endometrial cells, facilitating their implantation (10). Another commonly held theory is that of coelomic metaplasia. In this theory, normal peritoneum or residual embryonic müllerian tissue is stimulated by exogenous or endogenous hormones and transforms into endometriosis. This theory is supported by cases of endometriosis found in patients with müllerian agenesis. Other theories include metastasis of endometrial cells through lymphatic or hematologic dissemination to distant sites of the body (10). With the exception of the spleen, endometriosis has been found in every site in the body including the brain and lymph nodes. Endometriosis may also be iatrogenic and has been found in trocar sites and incisional scars. Almost 50% of bladder endometriosis is found in women with a prior cesarean section (14). Finally, there is a genetic component to the disease; first-degree relatives have a 7% risk of endometriosis (15). In summary, although there are numerous theories, the true pathogenesis of endometriosis is still unknown, complex, and likely a combination of all the above processes. In the following manuscript, we describe the two most common types of extragenital endometriosis: genitourinary and gastrointestinal, and discuss the diagnosis and management of this disease.

## GASTROINTESTINAL ENDOMETRIOSIS

### Diagnosis

**Figure 1:**
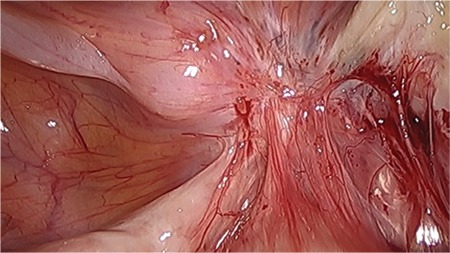
Endometriosis involving the rectum

**Figure 2:**
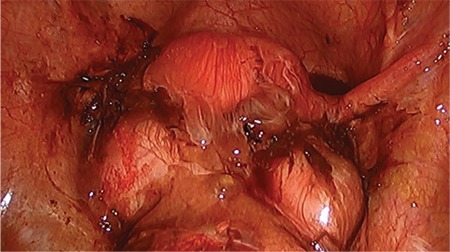
Ruptured bilateral endometriomas causing complete obliteration of the posterior cul-de-sac

Gastrointestinal endometriosis is most commonly found on the rectosigmoid colon (90% of cases of intestinal endometriosis), followed by the rectum, ileum (12%), appendix (8%), and cecum (6%) (2,16) (Figures 1 and 2). There have also been case reports of endometriosis found on the transverse colon (17) and stomach (18). Gastrointestinal endometriosis should be suspected in patients who report deep dyspareunia, dyschezia, catamenial diarrhea, hematochezia, constipation, pain with sitting, and pain radiating to the perineum. Lesions of the enteric nervous system may cause nausea, vomiting, and bloating if they involve Aurbach’s plexus, Meisner’s plexus, or the interstitial cells of Cajal (19).

### Medical management

Medical therapy is the first-line treatment for bowel endometriosis because of the potential morbidity of surgical treatment. However, it may not provide long-term improvement, and these patients may eventually require surgical management. It may also be used in patients who are not surgical candidates or prefer to avoid surgery. It has been shown to be effective by significantly alleviating symptoms in patients with less than 60% bowel stenosis (20). Hormonal suppression may also be used pre-operatively to reduce the disease burden, or post-operatively to prevent disease progression and recurrence (21). Medical treatment options are the same as those used for pelvic endometriosis, and include progesterone only, estrogen-progesterone combination contraceptives, the Mirena intra-uterine device, gonadotropin-releasing hormone (GnRH) agonist with or without add-back therapy, aromatase inhibitors, and danazol (22). Although medical management can significantly improve symptoms in 53% of patients, 33% of patients (23) eventually opt for surgical management after a 12-month period owing to persistent symptoms.

### Surgical management

**Figure 3:**
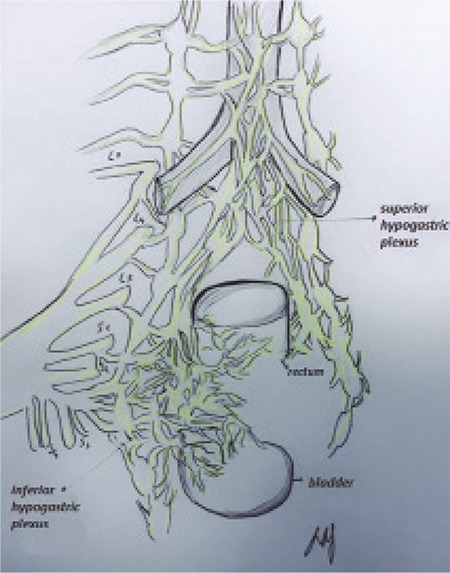
Innervation of the bowel

Surgical management is recommended for symptomatic patients who are refractory to medical therapy, or in whom medical therapy is contraindicated. The recommended surgical approach depends largely on the location and number of lesions, size and depth of the lesion, degree of circumferential involvement, and the presence or absence of stricture (24,25,26). Of these, location is the most important in dictating surgical procedure choice. We recommend a laparoscopic approach, whenever feasible, due to the numerous advantages of video-assisted laparoscopy with or without robotic assistance (VALRA) over laparotomy, including lower blood loss, less adhesion formation, less postoperative pain, shorter hospital stays, fewer postoperative complications, and improved fertility rate (27,28,29,30,31,32). The optimal surgical approach to lesions involving the rectum and sigmoid colon is controversial. The most important factor is the location of the lesion because lower rectal lesions require extensive dissection of the retro-rectal space and pelvic sidewall. Dissection of this space risks injury to the superior and inferior hypogastric plexuses, parasympathetic and sympathetic nerves, and vasculature (Figure 3). Injury to these structures may result in bowel, bladder or sexual dysfunction. Generally, the lower the lesion, the higher the risk of complications. Other complications involving dissection of the retrorectal space may include fistula, anastomotic leakage or stricture, bowel obstruction, bowel perforation, bowel and bladder incontinence, bowel stenosis or ischemia, bleeding, infection, constipation, and urinary retention (28,29,32,33,34,35,36,37). While some advocate complete resection of bowel endometriosis because of the risk of recurrence, we recommend a conservative surgical approach with shaving excision, particularly for lesions located 5-8 cm from the anal verge (38). Although shaving excision is associated with higher recurrence rates, there are multiple reported cases of bowel endometriosis recurrence even after radical segmental resection, possibly due to occult microscopic endometriosis, which can be found in 15% of specimen margins (39,40). Roman et al. (32) estimated that to prevent the risk of a single recurrence of bowel endometriosis that would necessitate repeat surgery, 23 patients would need to be treated initially with segmental bowel resection. In our experience, shaving excision provides high success rates with the lowest complication rates.

### Techniques

The three surgical approaches, from least conservative to most aggressive, are described below (Video 1. Bowel Endometriosis: Safe Endoscopic Excision of Deep Infiltrating Extragenital Endometriosis, https://www.youtube.com/watch?time_continue=2&v=inUVHCLzrQQ, Feb 8, 2107):

### Shaving excision

Shaving excision is the most conservative approach to surgical management of bowel endometriosis. It is performed through progressive layer-by-layer removal of diseased bowel until underlying healthy tissue is reached. It can be performed via ablation or excision. The aim is to remove as much endometriosis and fibrosis as possible, and restore a normal anatomic architecture without entering the bowel lumen (41,42,43,44,45,46). This technique is associated with lower complication rates compared with the other two techniques (44,45,46,47), and is recommended for lesions below the sigmoid colon owing to the abundant vasculature and nervous plexi supplying the lower rectum (38).

## DISC EXCISION

Disc excision removes the full thickness of the diseased portion of bowel, and is indicated in patients with DIE of the bowel, which may involve the mucosa (48,49,50). The defect is repaired either by suturing or stapling (28,41,43,48,49,51,52,53,54,55). To be a candidate for disc excision, the patient’s lesion must be smaller than 3 cm and involve less than 1/3 of the circumference of the bowel, in order to prevent stricture and stenosis (31). Suturing should be performed perpendicular to the long axis of the bowel to avoid shortening the length of bowel. Disc excision has good outcomes, with less risk of postoperative complications compared with segmental resection, but more than shaving excision (24,25,26,38,46,48).

### Segmental resection

Segmental bowel resection has been reported in the medical literature since 1907. It is indicated for multifocal or obstructive lesions, lesions larger than 3 cm, or lesions involving more than one-third of the bowel lumen (27,49,56,57,58). It involves complete resection of the diseased segment of bowel with primary end-to-end or side-to-side anastomosis. It can be performed via laparotomy or laparoscopically by surgeons trained in advanced laparoscopic techniques. Given the potential complications, it should be reserved for patients who fail medical management, or those with persistent symptoms after more conservative surgery. It is important to ensure well-vascularized and tension-free anastomoses to minimize the risk of anastomotic leakage (2,37). Recently, injection of intravenous (i.v) indocyanine green has been proposed as a method to ensure well-vascularized margins. Surgeons can immediately visualize perfusion to the colon at the site of re-anastomosis at the time of surgery (38).

### Location

Lesions can be categorized into four locations:

1) Above the sigmoid,

2) Sigmoid,

3) Rectosigmoid, and

4) Rectal.

### Lesions above the sigmoid colon

Lesions above the sigmoid colon generally do not require extensive retroperitoneal dissection. Segmental or disc resection is performed preferentially along the antimesenteric surface of the bowel to spare the vascular and nervous plexuses located in the mesentery. Lesions of the small bowel, ileocolic region, right hemicolon, and appendix are removed via segmental resection (2). The appendix should be inspected carefully for endometriosis and removed if abnormal because endometriosis commonly coexists on the appendix. There may be a benefit to removing the appendix even if it appears normal due to the high incidence of occult appendiceal endometriosis (38,59,60,61).

### Lesions along the sigmoid colon

Segmental resection at or below the sigmoid should be avoided whenever possible due to the risk of postoperative morbidity associated with dissection of the retrorectal space (34,62,63). Even disc excision involving dissection laterally and posteriorly risks injury to the nerves and vasculature, potentially leading to anastomotic leak, and bowel and bladder dysfunction necessitating long-term self-catheterization or colostomy. We prefer shaving excision for lesions at or below the sigmoid colon. When this technique is used, a thorough evaluation of the bowel thickness should be performed to assess the bowel wall integrity and thickness, and significant defects reinforced with suture. Disc excision or segmental resection may be performed, if indicated (38).

### Lesions along the rectosigmoid colon

Surgeons must exercise extreme caution when excising lesions at the level of the rectosigmoid colon; segmental resection at this level is often approached through the rectum or vagina (27,64,65,66). Segmental resection of lesions in this location often requires significant lateral mobilization and dissection of the retrorectal space. We recommend shaving excision, even in cases with lesions larger than 3 cm unless the patient has failed prior surgical management. Disc excision is possible, but must be performed with caution (38).

### Lesions along the rectum

We exclusively recommend shaving excision for lesions in this region except in cases of acute obstruction due to the extensive dissection required, which will inevitably compromise the surrounding neurovascular structures (38).

## URETERAL ENDOMETRIOSIS

### Diagnosis

**Figure 4:**
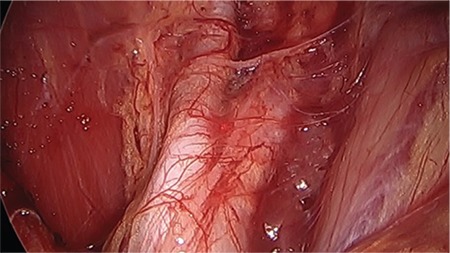
Extrinsic endometriosis of ureter causing ureteral obstruction and hydroureter

**Figure 5:**
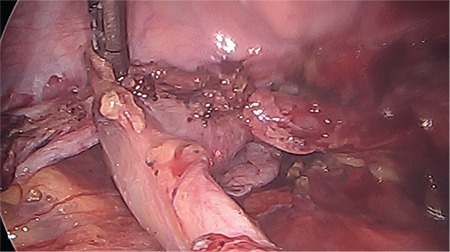
Intrinsic endometriosis of ureter causing stricture and hydroureter

The most common sites of urinary tract endometriosis are the bladder, ureter, and kidneys, with a ratio of 40:5:1, respectively (67,68,69). Ureteral endometriosis can be difficult to diagnose because it is asymptomatic in over 50% of patients (70,71,72). This can be dangerous because it can cause silent kidney loss if it results in ureteral stricture and obstruction (71,72). If symptoms are present, patients usually present with the usual symptoms of pelvic endometriosis: dysmenorrhea, pelvic pain, dyspareunia. Few present with specific urinary tract symptoms (e.g., flank or abdominal pain, dysuria, hematuria) (70). Ureteral endometriosis can be divided into extrinsic or intrinsic disease. Extrinsic, or superficial disease, is 4-5 times more common than intrinsic disease (68,70,73). It is caused by superficial endometriosis of the serosa of the ureter that compresses the ureter from fibrosis of the overlying peritoneum (Figure 4). It may also be caused by a large endometrioma adherent to the pelvic sidewall causing compression of the ureter. Intrinsic disease invades deeply into the ureteral wall, muscularis, or mucosa, and requires pathologic confirmation (Figure 5). It accounts for 20% of ureteral endometriosis. It is more commonly symptomatic with some patients reporting cyclic flank pain, but still less than 15% of patients will present with cyclic hematuria (70).

### Imaging

There are numerous imaging modalities that may be used to diagnose ureteral endometriosis: computed tomography urogram, magnetic resonance imaging (MRI), i.v pyelogram/retrograde pyelogram (RVP), and transvaginal ultrasound. Ultrasound is best for detecting ovarian and bladder endometriotic lesions, and frequently fails to detect ureteral endometriosis (74). It is highly dependent on the skill and expertise of the sonographer. A renal ultrasound is indicated to evaluate for hydronephrosis in women with suspected urinary tract endometriosis, and may be used to measure the degree of hydroureter and point of constriction (75). i.v pyelogram can be particularly useful in diagnosing intrinsic disease and to evaluate the degree and level of obstruction. It can also be used to evaluate ureteral patency after surgical treatment. If i.v contrast is contraindicated, a RVP can provide the same results. MRI is a sensitive modality in cases of DIE. However, a 2016 Cochrane review concluded that no imaging modalities were superior to surgery in the diagnosis of endometriosis, although it notably excluded bladder and ureteric endometriosis in the study. The goal of treatment is to relieve ureteral obstruction and compression from endometriosis, thus preserving renal function (73,76). Although a pre-operative renogram is unable to predict the return of kidney function after surgical decompression of obstruction, it may be considered if hydronephrosis or the hydroureter is present. A kidney is considered salvageable if more than 10% of renal function remains. If the glomerular filtration rate is less than 10%, a nephrectomy may be considered after consultation with a urologist.

### Medical management

Medical management of ureteral endometriosis is not recommended due the serious permanent sequelae of disease progression, high risk of failure, and risk of recurrence (77). It can, however, be considered for mild disease, or if a patient is not a candidate for surgery, and after careful discussion with the patient regarding the risks/benefits of conservative therapy. It is contraindicated when ureteral obstruction or hydronephrosis is present due to the risk of kidney loss. The goal of treatment is to induce regression of endometrial tissue, prevent endometriosis proliferation, and progression to ureteral obstruction.

### Surgical management

**Figure 6:**
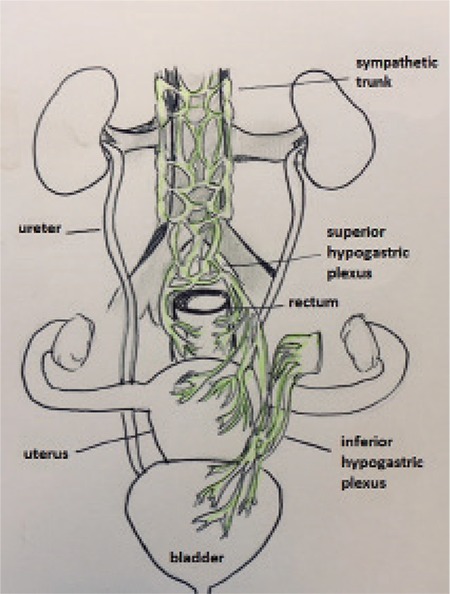
Innervation of the ureter

Laparoscopic treatment of endometriosis with complete excision of fibrotic lesions is the treatment of choice (67,71,78,79,80,81,82,83). VALRA offers numerous advantages over laparotomy, in particular improved visualization and magnification of endometriotic lesions, less blood loss, and less adhesion formation. The carbon dioxide laser or plasma jet energy with hydrodissection is our preferred technique, as it is much more precise and is associated with less thermal spread compared to electrocautery. This, in turn, may prevent unintentional injury to the ureters and surrounding vasculature (Figure 6). The retroperitoneum is injected with saline or dilute vasopressin using a laparoscopic needle to lift the peritoneum away from the underlying structures. The ureters and vessels are protected from the laser beam because the laser beam is unable to penetrate fluid. A peritoneal incision is made in an unaffected area using the laser to create a 0.5 cm opening. Lactated ringers or normal saline solution is then injected into this space by inserting the suction irrigator tip into the peritoneal opening. The endometriosis lesion is excised with 1-2 cm margins and the overlying peritoneum is peeled away while using the suction irrigator as a backstop to the laser (46) (Video 2: Laparoscopic Treatment of Genitourinary Endometriosis with and without Robotic Assistance, https://www.youtube.com/watch?v=zFdWu-wvM2E, Feb 8 2017). Laparoscopic ureterolysis has been shown to be successful in 90% of cases of hydroureter caused by ureteric endometriosis. The type of procedure performed depends on the location and depth of the lesion. For intrinsic disease or if hydroureter persists after ureterolysis, ureteral resection is indicated (68). If the lesion is located in the lower third of the ureter, close to the bladder, a ureteroneocystostomy with or without Psoas hitch may be performed. A larger distance may require a Boari flap, ileal interposition, or autotransplantation (78). Lesions in the middle or upper third of the ureter may require a ureteroureteral anastomosis (67,70,78,84). It is important to ensure that all anastomoses remain free of tension to prevent leakage and fistula formation (83).

## BLADDER ENDOMETRIOSIS

In contrast to ureteral endometriosis, bladder endometriosis is usually symptomatic. Patients may present with dysuria, hematuria, suprapubic pain, urinary urgency and frequency (70,79). Fortunately, it is usually associated with less morbidity compared with ureteral endometriosis. However, if an endometriosis lesion implants at the ureteral orifice and causes obstruction, it can also theoretically lead to hydroureter, hydronephrosis, and eventual kidney failure. Ultrasound and MRI both have high specificity for the detection of bladder endometriosis for lesions larger than 3 cm (7). Cystoscopy can identify deeply infiltrating endometriosis, seen as bluish lesions in the bladder mucosa. It can also estimate the distance from the lesion to the ureteral orifice, which is important in counseling patients on the risk of reimplantation if the lesion is less than 2 cm from the ureteral orifice. i.v pyelogram may show a filling defect if a bladder endometriotic lesions is present.

### Medical management

Medical therapy is generally considered a temporary solution because symptoms invariably return after discontinuation of medical therapy, and must be continued until menopause. It is preferred if asymptomatic, or if the lesion lies very close to the trigone, because excision can cause postoperative neurogenic bladder and retrograde bladder reflux due to disruption of the nerve and blood supply (83). Medical therapy options are the same as those for pelvic endometriosis. GnRH agonists can cause superficial bladder lesions to regress and have been found to be more effective than combined oral contraceptives. The typical treatment length is limited to 6 months because of bone loss with prolonged GnRH agonist use. If the symptoms persistent despite conservative medical management, surgical excision can be considered.

### Surgical management

Laparoscopic surgical management is the treatment of choice (11,85). The specific procedure depends on the location and depth of invasion. For superficial bladder lesions or extrinsic disease, either excision or fulguration is acceptable (26,82). Excision is preferred to remove the entire lesion, reduce the risk of recurrence, for pathologic confirmation, and to rule out malignancy (83).

For detrusor muscle involvement, bladder endometriotic lesions or intrinsic disease, a segmental bladder resection may be required (70). Fortunately, laparoscopic segmental bladder resection usually heals well owing to the abundant vascularization. It provides the best results in terms of symptomatic relief, disease progression, and recurrence risk. Laparoscopic excision should be performed concurrently with cystoscopy to ensure correct margins and complete excision. One- or two-layer closure using barbed sutures for bladder closure showed improved efficacy and more secure wound closure compared with monofilament sutures in one study (86). It is important to ensure a water-tight closure to prevent fistula or uroma formation. Ureteral stents should be placed when the lesion is near the trigone or within 2 cm of the ureteral orifice to maintain ureteral patency during the healing process. A ureteroneocystostomy may be required if the lesion is less than 2 cm from the ureteral orifice or close to the interureteric ridge (79). (Video 3: Robotic Assisted Laparoscopic Segmental Bladder Resection for Infiltrative Endometriosis, https://www.youtube.com/watch?time_continue=2&v=VPjCMhJoxuI, Sept 12, 2016). A catheter should be placed and left in situ for 1-2 weeks to decompress the bladder. A routine cystogram should be performed prior to catheter removal to confirm there is no urine leakage (73,75,87).

### Deeply infiltrating endometriosis

DIE is defined as the presence of endometriosis lesions more than 5 mm below the peritoneal surface. DIE is commonly found in the posterior cul-de-sac, the most dependent location for menstrual blood to collect. DIE behaves differently compared with superficial peritoneal endometriosis and has been found to express a higher level of invasive mechanisms such as matrix metalloproteinases and activins (88), which allow it to resist the suppressive effects of peritoneal fluid (89,90). An Allen-Masters peritoneal defect may act as a pathway for DIE in rectovaginal endometriosis. Physical exam findings may be particularly helpful at the time of menstruation when lesions may be more inflamed, tender, and palpable. Findings may include palpable nodules or thickening of the uterosacral ligaments, uterus, vagina, or rectovaginal septum (21). The presence of palpable nodules on rectovaginal exam indicates likely DIE and should prompt an evaluation for gastrointestinal and genitourinary endometriosis because these are more commonly found in patients with coexisting DIE (68). Transvaginal ultrasound is the initial imaging of choice for the diagnosis of DIE. A meta-analysis by the IDEA group showed transvaginal sonography had a high sensitivity and specificity for diagnosis of uterosacral, rectovaginal, vaginal, and bladder endometriosis (75). Renal ultrasound is also recommended in cases of DIE to evaluate for hydroureter/hydronephrosis. Much like gastrointestinal or genitourinary endometriosis, medical treatment of DIE is often ineffective and temporary - symptoms tend to recur once therapy is discontinued (7).

### Nerve-sparing surgery

Many surgical complications are a result of disruption of the superior and inferior hypogastric nerve plexus, which may be difficult to avoid in cases of DIE, which frequently involve these structures (91). Dissection in this area and disruption of this nerve supply may cause worsening or new-onset urinary dysfunction, such as urinary retention, dysuria or incontinence (36,92). Nerve-sparing surgery has been proposed as a method to reduce the risk of injury to these vital structures (58,93). The Tokyo method is a procedure in which the surgeon separates and ligates the vascular portion of the cardinal ligament while preserving the branches of the pelvic splanchnic nerves (94). Kockel described a technique sparing pelvic ligaments containing peripheral pelvic nerves and using liposuction to expose the autonomic pelvic nervous system. Possover (95) described a technique using electrostimulation to identify and avoid the parasympathetic pelvic nerves, known as the LANN technique. In a prospective study by Ceccaroni et al (58), which compared laparoscopic resection vs. laparoscopic nerve-sparing surgery, the authors found a significant reduction in bladder, rectal, and sexual dysfunction with nerve-sparing techniques. Furthermore, both groups had similar rates of intra-operative complications (58).

### Risk of malignant progression

The risk of developing endometriosis-associated neoplasm is estimated to be up to 1%, with 25% of these cases involving extra-ovarian tissue (96). Endometriosis is associated with an increased risk of endometrioid and clear cell adenocarcinoma (97,98). Thus, excision of endometriosis has the additional benefit of potentially reducing the risk of progression to cancer.

## CONCLUSION

Extragenital endometriosis is relatively rare, but may be more common than many realize due to its difficulty in diagnosis. Thus, it is important to thoroughly evaluate the patient for gastrointestinal and genitourinary endometriosis, especially if DIE is present on physical exam. When advanced stage disease is suspected, imaging with ultrasonography, MRI, or i.v pyelogram is necessary. Ultrasound is sufficiently sensitive for diagnosis of pelvic DIE and bladder endometriosis; however, MRI or i.v pyelogram is often needed to evaluate the intestines or ureter. Alternatively, a kidney ultrasound is indicated in cases of suspected genitourinary endometriosis to look for hydroureter or hydronephrosis. For gastrointestinal endometriosis, medical management should be tried first due to the risk of postoperative complications associated with injury to the nervus plexi supplying the lower rectum. Surgery should be considered as a second-line treatment only if medical management fails, and a conservative approach with shaving excision is preferred over disc or segmental excision when indicated. The recommended surgical approach depends largely on the patient’s symptoms, the location of disease, and the size and depth of the lesion. Nerve-sparing surgery, such as the Tokyo method, Kockel or LANN technique have been suggested to reduce the risk of nerve damage and subsequent complications, resulting in bowel, bladder, or sexual dysfunction. VALRA is recommended over laparotomy owing to the numerous advantages of laparoscopy, such as less postoperative pain, lower blood loss, less adhesion formation, magnification of lesions, and faster postoperative recovery. For ureteral endometriosis, complete surgical excision is preferred due to the permanent and serious sequelae of silent kidney failure associated with disease progression to ureteral stricture and obstruction. For bladder endometriosis, a trial of medical therapy may be appropriate if the patient is symptomatic because bladder endometriosis is not associated with kidney failure unless the lesion is located near the ureteral orifice and is causing obstruction. Medical management is usually temporary, less effective, and best used pre-operatively to reduce the disease burden, if the patient is unsuitable for surgery, or for post-operative hormonal suppression. Surgical excision and complete removal of lesions has the additional advantage of preventing malignant transformation because endometriosis has been associated with clear cell and endometrioid ovarian cancer.
